# Nine-year comparison of presentation and management of acute coronary syndromes in Ireland: a national cross-sectional survey

**DOI:** 10.1186/1471-2261-5-5

**Published:** 2005-02-11

**Authors:** Frank Doyle, Davida De La Harpe, Hannah McGee, Emer Shelley, Ronán Conroy

**Affiliations:** 1 Department of Epidemiology and Public Health Medicine, Royal College of Surgeons in Ireland, 123 St. Stephen's Green, Dublin 2, Ireland; 2 Department of Psychology, Royal College of Surgeons in Ireland, 123 St. Stephen's Green, Dublin 2, Ireland

## Abstract

**Background:**

Shorter time to treatment is associated with lower mortality in acute coronary syndromes (ACS). A previous (1994) survey showed substantial delays for acute myocardial infarction (AMI) in Ireland. The present study compared current practice with 1994 and surveyed acute coronary syndromes as a more complete contemporary evaluation of critical cardiac care than assessing AMI alone.

**Methods:**

Following ethics committee approval, all centres (N = 39) admitting acute cardiac patients to intensive/coronary care unit provided information on 1365 episodes. A cross-sectional survey design was employed.

**Results:**

Since 1994, median hospital arrival to thrombolysis time was reduced by 41% (76 to 45 minutes). Thrombolysis was delivered more often in the emergency department in 2003 (48% vs 2%). Thrombolysis when delivered in the emergency department was achieved faster than thrombolysis delivered in intensive/coronary care (35 mins v 60 mins; z = 5.62, p < .0001). Suspected AMI patients who did not subsequently receive thrombolysis took longer to present to hospital (5 h vs 2 h 34 mins; z = 7.33, p < .0001) and had longer transfer times to the intensive/coronary care unit following arrival (2 h 17 mins vs 1 h 10 mins; z = 8.92, p < .0001). Fewer confirmed AMI cases received thrombolysis in 2003 (43% vs 58%). There was an increase in confirmed cases of AMI from 1994 (70% to 87%).

**Conclusions:**

Substantial improvements in time to thrombolysis have occurred since 1994, probably relating to treatment provision in emergency departments. Patient delay pre-hospital is still the principal impediment to effective treatment of ACS. A recent change of definition of AMI may have precluded an exact comparison between 1994 and 2003 data.

## Background

Ireland has one of the highest mortality rates from cardiovascular disease in the European Union [1]. Acute coronary syndrome (ACS) is a major portion of cardiovascular diseases. ACS includes unstable angina and both persistent-ST-segment elevation and non-ST segment elevation acute myocardial infarction (AMI) [2-4]. Thrombus formation is the primary reason for myocardial infarction [5]. This usually occurs after a complex interaction between coronary atherosclerosis, plaque rupture and platelet activation. Thrombolysis is an appropriate treatment for thrombus formation in ST-elevation AMI and when delivered in a 'timely' manner, preferably within 6 hours but including up to 12 hours after symptom onset [6,7], can significantly reduce morbidity and mortality from AMI. Each hour of time saved can lead to a decrease of about 1.6 deaths per 1000 patients treated [7]. Therefore, shortening of time to treatment for AMI patients is an important life-saving goal for health services [6].

International guidelines, for example those from the European Society of Cardiology [8,9] and the British Heart Foundation [10], have proposed a 'call-to-needle' time of 90 minutes for thrombolysis administration. The National Service Framework (NSF) in the United Kingdom (UK) further reduced the recommended time in 2000 with a proposed 'call-to-needle' time of 60 minutes [11]. Furthermore, eligible patients should be thrombolysed within 30 minutes of arrival at hospital [9]. In Ireland, the 1994 national census found a median time to treatment of 4 hours 30 minutes [12]. This compares unfavourably to more recently recorded median treatment times in other countries, e.g. 2 hours 45 minutes in the UK [13] or 2 hours 54 minutes in Switzerland [14]. Approximately 50% of AMI deaths in the community occur within two hours from the onset of symptoms [15]. Early management of AMI patients with thrombolysis significantly reduces morbidity and mortality [16]. Time to treatment can be shortened by thrombolysing patients in the emergency department prior to transfer to intensive/coronary care. This strategy has lead to significant reductions in delay [17].

Since 1994, there has been no examination of time to treatment and the extent to which international guidelines for treatment of AMI are being achieved in Ireland. This study assessed the presentation and management of a national cohort of suspected ACS patients admitted to intensive/coronary care units (I/CCUs) in all 39 Republic of Ireland hospitals providing such care. We decided to extend the range of patients assessed from those with suspected AMI (as was the case in previous surveys [12,18]) to all suspected ACS patients, to profile the pool of possible patients presenting from which testing determines eligibility for reperfusion therapy. Differing proportions of reperfusion-eligible patients over time or centre, alongside the absolute number of patients presenting, may influence the speed of management of eligible patients. Also, some treatments for other ACS (e.g. unstable angina) can be similar to treatment for AMI, depending on the severity of the event [19] (e.g. angioplasty treatment for unstable angina). Changes in the definition of AMI in recent years have lead to an increase in the proportion of diagnosed AMI patients and a decrease in the proportion of patients diagnosed as having unstable angina [20-22]. which may preclude exact comparison to previous findings. Where possible we compare data to results from 1994.

## Methods

### Sample

All Irish centres admitting suspected ACS patients to I/CCU (N = 39) agreed to participate following relevant ethics approval [23]. Data collection was conducted from January to October 2003. Four hospitals had not recruited 25 suspected AMI patients by the study cut-off date. Suspected acute coronary syndrome (ACS) patients admitted to I/CCU were recruited. Staff were provided with the consensus definition of ACS as agreed in 2000 by the Joint European Society of Cardiology/American College of Cardiology Committee [4]. This definition uses enzyme (troponin) change as a marker of myocardial necrosis. The survey was of suspected ACS and the main focus was on how patients with suspected ACS are treated in the early phase of their hospital admission. Therefore the admission diagnosis was used to categorise patients (e.g. if a patient was admitted to I/CCU with a diagnosis of 'chest pain – query AMI', they were listed as suspected AMI for the purposes of this study; if patients were admitted with suspected ACS or suspected unstable angina, they were categorised as 'other ACS'). Data on successive admissions were audited anonymously from hospital charts. Participating hospitals recruited all consecutive suspected ACS patients, until 25 suspected cases of AMI had been admitted to I/CCU. Data on a total of 1365 episodes were collected (935 suspected AMI and 430 suspected other ACS admitted contemporaneously). Data collected assessed demographic details, clinical history, risk factors, presentation and management profile. Eligible patients were also approached to participate in a follow-up survey (results to be reported elsewhere).

### Analysis

Analysis was conducted on the data using STATA/SE 8.0. Mann-Whitney U tests were used to test for significance between treatment times, χ^2 ^was used for categorical variables, and t-tests were used for continuous variables. Results from 1994 are reported, but significance tests were not conducted between 1994 and 2003 data (as raw data from 1994 was unavailable). Total time to treatment was defined as follows: symptom onset to reperfusion (thrombolysis or direct infarct angioplasty). Inpatient and other hospital transfer times were not included in the analysis for 1994 or 2003 samples.

## Results

### Baseline characteristics

The sample consisted of 1365 episodes, 935 suspected AMI and 430 suspected other ACS patients. The gender breakdown has been described elsewhere (manuscript submitted for publication). The overall mean age was 64 years (std dev = 13; median = 65; range = 20–100 yrs). Admission characteristics are shown in table 1.

**Table 1:** Comparative demographic and admission profile in 1994 and 2003

**Demographic**	**1994 **(N = 950 suspected AMI)	**2003**		
		
		Suspected AMI (N = 935)	Suspected Other ACS (N = 430)	Combined (N = 1365)
**Mean age (years) **(mean) (std dev)	-	66 (13)	61*** (14)	64 (13)
Men	64 (12)	64 (13)	60*** (13)	63 (13)
Women	69 (11)	71 (13)	64*** (14)	69 (13)

**Referral source (%)**				
Primary care physician	69	53	55	53
Self	24	39	33	38
Other	7	8	12	9

**Admission mode to hospital (%)**				
Ambulance	46	48	38**	45
Car (passenger)	42	42	43	42
Car (driver)	8	6	12***	8
Other	4	4	7	5

**Distance from hospital at symptom onset**				
Median (range) miles	9 (0–165)	9 (0–80)	8 (0–150)	8 (0–150)

**Previous CHD history (%)**				
AMI	24	16	31***	21
Unstable angina	14	14	28***	19
Coronary artery bypass graft	4	5	13***	7
Percutaneous coronary intervention	2	6	18***	10

(*p < .05, **p < .01, ***p < .001)

The demographic profile of patients admitted for suspected AMI in 2003 appears similar to those admitted in 1994. Other ACS patients differed from suspected AMI patients in 2003 in the following aspects: they were younger, less likely to be admitted by ambulance, more likely to drive themselves to hospital, and had a higher prevalence of previous ACS and coronary interventions.

### Thrombolysis

**Figure 1:**
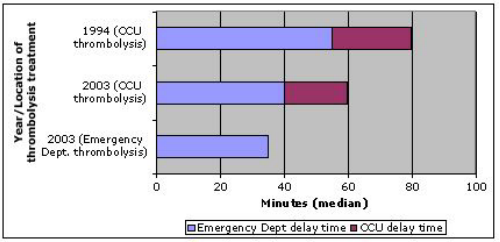
Thrombolysis administration locations and treatment times in 1994 and 2003

Both location of, and speed of administration of thrombolysis have changed considerably in a positive direction since 1994 (Figure 1). In 1994, 38% of suspected AMI and 58% of confirmed AMI patients received thrombolysis, which occurred in I/CCU (96%), emergency department (2%) or other location (2%). In 2003, 41% of suspected AMIs and 44% of confirmed AMIs were thrombolysed in I/CCU (48%), emergency department (48%) or other location (4%). A further 4% of suspected AMIs received direct infarct angioplasty.

Emergency department delivered thrombolysis occurred in a significantly shorter time (median 35 minutes) than I/CCU administered thrombolysis (median 60 minutes) in 2003 (z = 5.62, p < .0001).

In 2003, 29% of those thrombolysed were treated within 90 minutes of calling for professional help. This rose to 42% and 62% within 2 and 3 hours respectively. On hospital arrival, 35% of patients were thrombolysed within 30 minutes, rising to 60% and 74% within 60 and 90 minutes respectively. Thirty-six per cent of hospitals thrombolysed 80% or more of patients in the emergency department while 56% of hospitals thrombolysed over 50% of patients in the emergency department.

### Time to treatment

Suspected AMI patients waited a similar length of time to get to hospital from onset of symptoms in 2003 as 1994 (Table 2). Symptom onset to hospital arrival time was significantly higher for patients admitted with suspected other ACS in 2003 (3 h 35 mins vs 4 h 39 mins, z = 2.99, p < .01).

**Table 2:** Median overall time to treatment for all patients in 1994 and 2003

**Treatment times**	**1994 Suspected AMI **(n = 950)	**2003**				
		
		*Thrombolysed AMIs (n = 382)*	*Suspected AMI – non-thrombolysed (n = 553)*	**All suspected AMI **(n = 935)	**Suspected other ACS **(n = 430)	**Total **(n = 1365)
Symptom onset to hospital	3 h 30 mins	*2 h 34 mins*	5 h 00 mins***	3 h 35 mins	4 h 39 mins**	3 h 56 mins
Call-to-thrombolysis	Unavailable	*2 h 20 mins*	*-*	2 h 20 mins	-	-
Hospital arrival to I/CCU	55 mins	*1 h 10 mins*	2 h 17 mins***	1 h 40 mins	2 h 43 mins***	1 h 55 mins
Hospital arrival to thrombolysis	76 mins	*45 mins*	*-*	45 mins	-	-
I/CCU admission to thrombolysis	25 mins	*20 mins*	*-*	20 mins	-	-

(*p < .05, **p < .01, ***p < .001)

Patients waited longer to be admitted to I/CCU in 2003, but received thrombolysis more quickly (45 mins v 76 mins) after hospital arrival. This represents a 41% decrease in time-to-thrombolysis since 1994. In 2003, total time to treatment for suspected AMI patients who received reperfusion was 4 hours 00 mins.

Patients with suspected AMI who were subsequently thrombolysed (thrombolysed AMIs) presented to hospital (2 h 34 mins vs 5 h, z = 7.33, p < 0.0001) and had a faster I/CCU transfer time (1 h 10 mins vs 2 h 17 mins, z = 8.92, p < 0.0001) than suspected AMI patients who were not thrombolysed (non-thrombolysed AMIs). Suspected other ACS patients also waited significantly longer than non-thrombolysed AMIs for hospital transfer to I/CCU (2 h 43 mins vs 2 h 17 mins; z = 2.128, p < 0.05).

In 1994, treatment times for patients referred by primary care physicians were significantly longer than those who self-referred (symptom onset to hospital arrival: primary care physician-referred 4 h 15 mins, self-referred 2 h 05 mins, p < 0.001). In 2003, primary care physician-referred suspected AMI patients also had a significantly longer pre-hospital delay (5 h vs 2 h 28 mins, z = 7.9, p < 0.001).

For suspected AMI patients, a previous experience of AMI made no difference to hospital presentation time (3 h vs 3 h 45 mins, z = 1.2, p > 0.05). There were no gender differences in pre-hospital delay time (3 h 53 mins for men vs 3 h 14 mins for women, z = 0.63, p > 0.05), or hospital arrival to thrombolysis time (45 mins for men vs 50 mins for women, z = 1.43, p > 0.05) for suspected AMI patients in 2003.

### Discharge

For suspected AMI patients, there was an increase in those patients diagnosed with myocardial infarction of 21% from 1994 to 2003, and a 10% reduction in the diagnosis of unstable angina (Table 3).

**Table 3:** I/CCU discharge diagnoses and hospital mortality (%)

**Discharge diagnosis**	**1994 Census (suspected AMI) %**	**CCU 2003 Survey**				
		
		*Thrombolysed AMIs (n = 382)*	*Suspected AMI – non-thrombolysed (n = 553)*	**Suspected AMI**	**Suspected Other ACS**	**Total**
Myocardial infarction	70	*97*	86***	91	19***	68
Unstable angina	14	*<1*	6***	4	47***	17
Other cardiac	9	*5*	11**	9	32***	16
Non cardiac	7	*1*	5**	3	17***	8
Mortality	11	*9*	*10*	10	1***	7

Patients may have more than one diagnosis

(*p < .05, **p < .01, ***p < .001)

In 2003, thrombolysed AMIs were more likely to be discharged as having myocardial infarction than non-thrombolysed AMIs, but were less likely to receive a discharge diagnosis of unstable angina, other cardiac or non-cardiac diagnoses. All suspected AMI patients in 2003 were more likely to be discharged as having had myocardial infarction and were more likely to die in hospital than suspected other ACS patients, but were less likely to have discharge diagnoses of unstable angina, other cardiac or non cardiac.

## Discussion

The present survey outlines the current presentation and management of ACS in Ireland. This study built on the previous research conducted in 1994, but also expanded its findings beyond suspected AMI patients to all suspected ACS patients. Pre-hospital patient delay remains stable and substantial, while a considerable reduction (41%) in time to thrombolysis from hospital admission has occurred since 1994. This can probably be attributed in large part to the relocation of thrombolytic administration to the emergency department, thereby reducing the 'door-to-needle' times in 2003. Similarly, treatment of AMI patients in emergency departments prior to transfer to I/CCU may account for longer I/CCU transfer times in 2003.

Significant progress has been made in the treatment of AMI patients who receive thrombolysis, which has yielded faster 'door-to-needle' times. Suspected AMI patients who received thrombolysis in the 2003 sample were treated more quickly than 1994 (median 45 mins v 76 mins).

The transfer of thrombolytic administration from the I/CCU (96% in 1994) to the emergency department (48% in 2003, with 48% administered in I/CCU) is probably the main reason for the decreased time to treatment for thrombolysed patients. The present survey found, for instance, that 56% of hospitals thrombolysed over half of their patients in the emergency department. It is not clear, however, that an additional shift of thrombolysis to the emergency department in the remaining hospitals would also result in a further reduction of 'door-to-needle' time. This is because some hospitals already adopt a 'fast-track' policy, where chest pain patients are admitted directly to CCU, bypassing emergency department assessment. Adopting a strategy of emergency department thrombolysis may have little or no effect in these cases.

Nonetheless, only 35% of patients received thrombolysis within 30 mins of hospital arrival, which compares unfavourably to other surveys (e.g. in England and Wales in 2003, the MINAP study found that over 80% of patients were thrombolysed within 30 mins of hospital arrival [24]). These comparisons must be interpreted with some caution however, since MINAP does not count cases which have a component of extra delay due to clinical reasons (i.e. patients presenting with contraindications to thrombolysis), and reports on all eligible STEMI cases treated within 30 mins of hospital arrival. Our analysis included all patients who eventually received thrombolysis, even those patients who developed ST-elevation some time after hospital arrival. While our results may not be completely comparable to other similar surveys, the overall message that thrombolysis is unsatisfactory both in absolute and comparative terms is clear.

Substantial pre-hospital and in-hospital delays were seen for non-thrombolysed AMIs. Suspected AMI patients who did not receive thrombolysis waited significantly longer for transfer to I/CCU than those who received thrombolysis. Indeed, this group of AMIs had comparable times to patients with suspected other ACS. These patients presented to hospital more slowly than thrombolysed AMIs. This may indicate the less severe symptoms of unstable angina and non-ST-elevation myocardial infarction. Also, recent changes in AMI definition have lead to increases in proportions of diagnosed AMI patients and decreases in proportions of patients diagnosed with unstable angina [20-22]. These changes probably preclude exact comparison with 1994 findings. Adding credence to this hypothesis was the large change in discharge diagnoses. Considering suspected AMIs, the proportion of patients diagnosed with myocardial infarction increased by 21% from 1994 to 2003, with a corresponding 10% reduction in unstable angina diagnoses. The reduction in 'non cardiac' discharges for suspected AMI patients (7% in 1994 to 3% in 2003), may be partially attributed to definition change [21]. Finally, the 14% decrease in thrombolysed confirmed AMIs may also be attributed to the definition change, with a larger portion of AMI patients being ineligible for thrombolysis in 2003.

Suspected AMI patients who did not receive thrombolysis took significantly longer from hospital arrival to arrive in I/CCU than did patients who received thrombolysis. Suspected other ACS patients presented to hospital in time frames similar to suspected AMI patients who did not receive thrombolysis, but were not admitted to I/CCU as quickly. The development of chest pain assessment units and other similar units (e.g. medical admission units), which involve the initial screening of chest pain patients to determine whether the pain is cardiac in origin prior to transfer to I/CCU, may have skewed the data in the current survey. A typical scenario may be that a patient currently arrives at hospital, without ST-elevation when assessed by ECG, and is assessed for some time in the chest pain assessment unit to either 'rule-in' or 'rule-out' ACS. In the past, such patients may have been admitted directly to I/CCU. In addition, these findings may reflect the triaging of patients in the emergency department, where patients labelled as suspected AMI were treated more quickly than those labelled as suspected other ACS. Patients can be triaged into groups reflecting the necessity for immediate treatment. Non-ST-elevation myocardial infarction and unstable angina patients may be detained for observation in the emergency department. It might also be that pressure for I/CCU bed places is more quickly resolved for the more acute ST-elevation AMI patients. However, further prospective observational research on this aspect of care is required.

Patient delay prior to hospital arrival is still the biggest impediment to improving treatment times for AMI and other ACS. Patients who were referred by primary care physicians had a significantly longer time to treatment than those who self-referred in both 2003 and 1994. Clearly, in the present system, although substantial improvements have been made since the mid-1990s, a 'call-to-treatment' standard of 90 minutes is not currently being met. The present survey found that 29% of patients were thrombolysed within 90 minutes of calling for professional help. Future research and resources need to focus on a reduction in pre-hospital delay factors by the services and professional groups concerned. A number of psychological studies have provided detailed examination of patient contributions to delayed help-seeking for AMI [25-27]. These highlight possibilities for intervention to reduce symptom onset to help-seeking times but caution against a simplistic public education campaign approach. Since general public advertising/education campaigns have little efficacy [25,26,28] and even a previous experience of AMI has no effect on pre-hospital call for assistance times, sophisticated strategies are needed to address this problem.

As regards reducing health services delay, one method is to authorise health professionals in the pre-hospital setting to administer thrombolysis. Ambulance personnel and primary care physicians are the two most obvious choices. The administration of thrombolysis by ambulance personnel has been shown to reduce time to treatment [29]. A recently completed study on thrombolysis administered by primary care physicians showed the practicalities of this approach in an Irish setting [30]. In the hospital setting, involving nurses in decision-making processes for thrombolysis has been shown to be effective in reducing treatment times [31,32]. The provision of more mobile coronary care units may also reduce delay times in rural areas [33].

The present study has highlighted the need for a national prospective registry of ACS. The value of such registries has been shown in other countries. For example, registries increase the use of appropriate reperfusion therapy, but to ensure this practice continues they need to be ongoing [34]. In the UK, MINAP [35] has contributed to an increase in the numbers of patients thrombolysed within recommended timescales. For example, following the publication of the NSF guidelines on CHD in 2000 [11], the proportion of patients receiving thrombolysis within 30 minutes of hospital arrival more than doubled (79% in 2002, compared with 38% pre-2000) [36]. Registries can also underscore the extent of adherence to international guidelines. The current Irish data highlight progress in some areas over a nine-year period but indicate that improvements need to occur in other aspects. A national registry can provide the continuous feedback needed to keep a focus on areas for improvement. The monitoring of guideline adherence should even be considered a part of optimal practice procedures [37].

## Conclusions

Significant progress in some aspects (e.g. time to thrombolysis) of care for suspected ACS has occurred, but there is still scope for improvement. A necessary goal is to increase the proportions of patients seen within the recommended time. Other aspects of ACS care also now require attention. Patient delay pre-hospital should remain the main focus of future research. The implementation of a national registry would allow resources to be focused on these aspects and facilitate routine health care monitoring.

## Competing interests

The author(s) declare that they have no competing interests.

## Authors' contributions

FD carried out the survey, analysed the data and drafted the manuscript. HM, DD & ES conceived of the study and participated in its design and coordination. RC participated in the study design and coordination, and provided statistical input on data analysis and interpretation. All authors provided critical input on manuscript drafts, and approved the final version.

## Pre-publication history

The pre-publication history for this paper can be accessed here:


